# Rapid scale-up of hospital-at-home services during wartime: lessons learned from the Sheba Medical Center experience

**DOI:** 10.3389/fmed.2026.1859075

**Published:** 2026-08-27

**Authors:** Anat Ekka Zohar, Boris Fizdel, Liat Aroshass, Liron Vaserman, Galia Barkai, Shani Edelman, Rachel Sarafraz, Eyal Leshem, Matan Peri, Asaf Biber, Yael Frenkel Nir, Gad Segal

**Affiliations:** 1 Sheba BEYOND, HaH arm and Virtual Hospital, Sheba Medical Center, Ramat Gan, Israel; 2 Faculty of Health Management, Bar-Ilan University, Ramat Gan, Israel; 3 Dina Recanati School of Medicine, Reichman University, Herzliya, Israel; 4 Gray Faculty of Healthcare Science and Medicine, Tel-Aviv University, Tel-Aviv, Israel; 5 General Hospital Management, Sheba Medical Center, Ramat Gan, Israel

**Keywords:** crisis, hospital at home, telemedicine, transformation, wartime

## Abstract

**Background:**

Sheba BEYOND, the telemedicine-governed hospital within the Sheba medical center includes a unique telemedicine-based service for internal-medicine patients suffering from acute illness since 2020, serving as a viable alternative to in-hospital stay. During March 2026, amid an Israeli war on two fronts, Iran and Lebanon, the urgent need to transfer over 1,900 (in-hospital) patients to underground facilities under continuous missile attacks necessitated immediate, massive fortification of our HaH services across multiple clinical disciplines.

**Methods:**

Sheba BEYOND qualified its HaH services for home hospitalization of dozens of patients from several clinical disciplines. Both its headquarters, in-house and external nursing services and physicians had to rapidly scale up. We did a descriptive analysis and comparison of HaH activities between wartime (March 2026) and the previous month (February 2026).

**Results:**

During March 2026, mean daily HaH admissions increased almost three-fold, from 2.11/day to 6.06/day, corresponding to a rate ratio of 2.88 (95% CI 2.15–3.86; *p* < 0.001). Patients admitted during wartime were younger than those admitted pre-war (mean age 63.1 ± 20.6 vs. 71.5 ± 16.4 years, *p* = 0.002), and the clinical scope expanded from internal medicine to include surgical, pediatric, cardiology, gynaecology, and oncology patients. Operational capacity expanded from 12 to 40 HaH beds, the nursing workforce increased through recruitment of 30 additional nurses, and geographic coverage expanded from a 30-km radius to nationwide service using internal and external nursing providers. Short-term clinical indicators did not show statistically significant worsening: transfers back to hospital were 6.8% vs. 2.1% (*p* = 0.095), repeat hospital admission within 14 days was 8.5% in both groups, median HaH length of stay was 3 days in both periods (IQR 2–5 in both; *p* = 0.295), and no patient died during active HaH care. Because of important differences in age and case mix, these unadjusted clinical comparisons should be interpreted cautiously.

**Conclusion:**

This wartime experience demonstrates that a telemedicine-led HaH service can be rapidly scaled in volume, geographic reach, and clinical scope during a hospital capacity crisis. The available data support the feasibility of rapid operational expansion and provide reassuring, but unadjusted, short-term safety indicators rather than definitive evidence of clinical equivalence.

## Introduction

### Hospital at home as a viable option for acutely ill patients

Hospital-at-Home (HaH) refers to the delivery of time-limited, hospital-level services in a patient's home as a substitute for traditional inpatient admission (admission avoidance) or as a means to complete an acute episode after early discharge. Over the last three decades, HaH has been evaluated across diverse health systems and clinical indications, with systematic reviews and meta-analyses generally demonstrating that, for appropriately selected patients, outcomes are comparable to or better than conventional hospitalization, including similar mortality and readmission rates, improved patient satisfaction, and potential cost savings (1–3). The accelerating maturity of telemedicine, remote patient monitoring, and command-center models has broadened the feasibility of HaH by enabling rapid escalation pathways, continuous clinical oversight, and integration of community-based diagnostics and therapeutics into home-based care pathways (4–6).

### Wartime context and rationale

Mass-casualty threats, infrastructure disruption, and sustained missile attacks create simultaneous pressures on hospitals: the need to protect patients and staff (e.g., relocation to fortified or underground wards), while preserving the capability to deliver acute care to new and existing patients. Emergency preparedness literature commonly describes surge management in terms of expanding or reallocating *staff*, *space*, *supplies*, and *systems* (the “4S” framework), coupled with rapid throughput measures such as early discharge, inter-facility transfers, and the use of alternate care sites (7, 8). In this context, HaH can be conceptualized as a clinically governed “distributed bed capacity” strategy: it substitutes for inpatient beds by shifting a subset of eligible patients to home-based, hospital-led care, while maintaining escalation pathways to hospital services when needed. During wartime, this approach may reduce dependence on vulnerable physical bed capacity and support continuity of care despite restricted access to on-site wards (9, 10).

### Sheba BEYOND

Sheba BEYOND is a virtual hospital within Sheba Medical Center and provides remote, telemedicine-governed services. Its telemedicine-enabled internal-medicine HaH service has operated since 2020 as a viable alternative to inpatient admission for selected acute conditions. Consistent with widely described HaH models, care is anchored in hospital governance and clinical accountability, typically combining protocolized medical evaluation and daily physician oversight through telemedicine protocols, in-home nursing care, point-of-care and hospital-based diagnostics, medication administration including intravenous therapies when clinically indicated, and remote monitoring with 24/7 escalation protocols when referral back to the hospital is needed. Eligibility decisions in HaH programs are generally driven by a triad of factors: clinical stability, diagnostic clarity, and a safe home environment with reliable communication and the ability to rapidly return the patient to hospital if deterioration occurs. These principles guided baseline operations prior to the wartime surge described in this manuscript. Previous studies and publications have established the safety profile of Sheba BEYOND across heterogeneous patient populations, including validations of our main telemedicine platforms and protocols (4, 5, 11–13).

## Methods

We report the rapid expansion of a hospital led HaH service during a wartime period requiring large-scale relocation of hospitalized patients to fortified (underground) facilities. The observation period spanned February 1, 2026, through March 31, 2026. Data sources included routine service operational records (daily census, referral sources, covered clinical disciplines, geographic service radius, staffing rosters) and clinical outcome tracking used for quality assurance. All data were retrieved after the approval of the Chaim Sheba Institutional Review Board (approval # SMC-D-3208-26) who waived the need for informed consent due to the retrospective nature of this study.

Outcomes were categorized as operational capacity outcomes and clinical safety indicators. Operational capacity outcomes included total HaH admissions, mean daily admissions, active bed capacity, number of clinical disciplines served, geographic coverage, and workforce expansion. Clinical safety indicators included transfer back to in-hospital care during the HaH episode, repeat hospital admission within 14 days after HaH discharge, and death during active HaH care. Repeat hospital admission was defined as any unplanned admission to a conventional hospital ward within 14 days after discharge from HaH, regardless of whether the patient subsequently returned to HaH care. Mortality during HaH was defined as death occurring while the patient was actively enrolled in HaH; deaths after transfer back to hospital were reviewed separately and were not counted as deaths during active HaH care unless explicitly stated. Comparative analyses were conducted between the pre-wartime baseline period (February 1–28, 2026) and the wartime period (March 1–31, 2026), defined according to HaH entry date. Daily admission rates were compared as count data using a rate ratio calculated from admissions per person-day of observation, with exact Poisson confidence intervals. Age was compared using Welch's *t*-test. Length of stay was compared using the Mann–Whitney U test. Categorical outcomes were compared using Fisher's exact test. Statistical significance was defined *a priori* as *p* < 0.05. Statistical analyses were performed using Python. Because the wartime cohort differed in age and clinical composition, clinical outcome comparisons were considered descriptive and unadjusted; the study was not designed to establish clinical equivalence between periods.

## Results

### Infrastructure and capacity expansion

**Figure 1:**
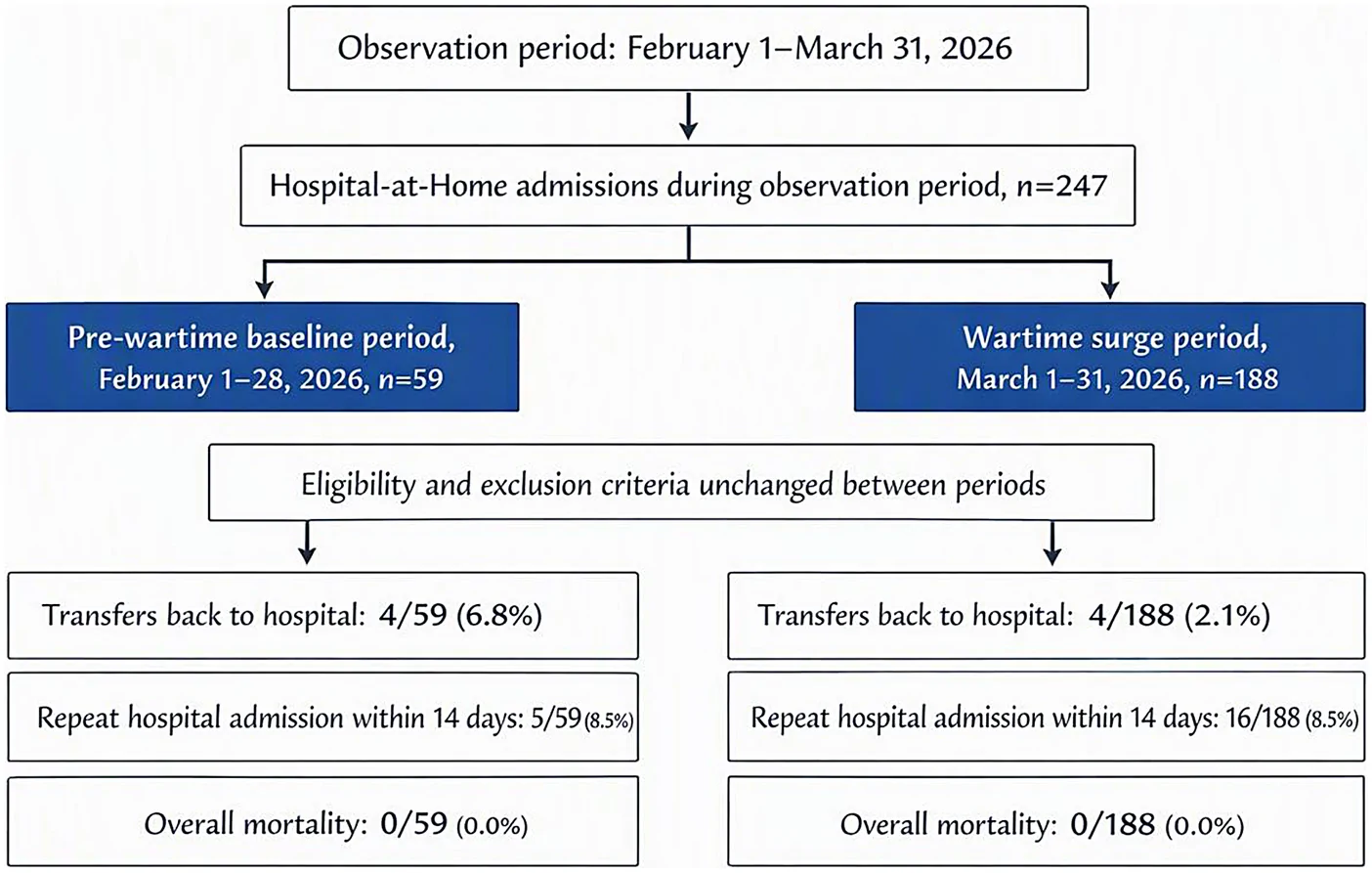
Patient flow diagram of Hospital-at-Home admissions included in the study.

The wartime response required a rapid, multi-domain expansion of our HaH capacity. Figure 1 present the patients' flow of hospital-at-home admissions included in this study. First, the service broadened its clinical scope beyond routine internal medicine admissions to include additional disciplines (surgical, pediatric, gynecologic, cardiology, and oncology), reflecting an urgent need to decant inpatient wards and preserve protected bed space for patients who could not be safely managed at home. Importantly, eligibility criteria and exclusion criteria did not change between wartime and pre-war period. Second, the service expanded its geographic reach from a local catchment area (previously limited to approximately a radius of 30 km around Sheba Medical Center) to nationwide coverage, enabled by a hybrid delivery model that combined centralized telemedicine oversight with both internal and contracted external nursing services for in-home visits. Third, workforce capacity increased substantially, with rapid onboarding and redistribution of clinicians and nurses to support a three-fold growth in daily admissions and active census, consistent with established surge principles emphasizing flexible staffing models and rapid role adaptation. Finally, the wartime expansion required strengthened infrastructures for triage, communication, and escalation, ensuring that home-treated patients could be promptly transferred to the emergency department or directly admitted to protected wards if clinical deterioration occurred, an essential safety requirement in HaH models and in telemedicine-enabled inpatient-at-home care.

### Training

Workforce expansion was implemented through rapid recruitment, redeployment, and focused training. To support growth from 12 active HaH beds to 24 beds and subsequently to 40 beds, nurses were recruited from clinics, inpatient departments, day-hospitalization units, and other hospital services, resulting in 30 additional nurses joining the HaH operation. Field nurses underwent a focused two-day training program followed by an overlapping supervised shift. Administrative staff were trained to support the call-center nurses and coordinate information flow among physicians, field nurses, families, patients, hospital leadership, the Care Control Center, and the Sheba BEYOND operational team. Medical staffing was reinforced by physicians from several departments, with operational contingency planning required when key personnel were called for military reserve duty.

### Equipment

Equipment and logistics were coordinated through a dedicated institutional resource forum established during the wartime response. Essential home-hospitalization equipment, including electrocardiography devices, infusion pumps, automated external defibrillators, glucometers, tablets, blood-pressure monitors, vehicles, and related supplies, was procured or redeployed within 2–3 days. The Sheba BEYOND command center was relocated to a protected alternative site, equipment stations were created, nursing work areas were converted for care-management activity, and patient rooms were repurposed for equipment storage. These logistical changes were coordinated by nursing leadership and the operations director. In parallel, business-intelligence reports and technological tools were adapted to support patient tracking, resource allocation, and quality monitoring during rapid scale-up.

Staff safety and continuity of care were additional operational considerations. Field teams continued home visits during missile alerts and periods of restricted movement. When alerts occurred during home visits, staff sheltered with patients and family members according to civil-defense instructions. This experience emphasized that HaH surge planning in conflict settings requires not only clinical and logistical protocols, but also explicit procedures for staff protection, transportation, communication, and psychological support.

### Management oversight

Management oversight was provided through cross-organizational coordination among hospital leadership, Sheba BEYOND leadership, nursing management, logistics, transportation, procurement, pharmacy, information technology, information security, the Sheba innovation arm, the Care Control Center, clinical teams, and the research team. This structure supported rapid decision-making regarding staffing, equipment allocation, information systems, escalation pathways, and quality assurance. The experience suggests that predefined governance mechanisms are essential for converting an established HaH service into a crisis-response platform.

### Comparison between pre-wartime and wartime periods

A total of 59 patients were admitted to the HaH service during February 2026 and 188 during March 2026. Comparisons are presented in Table 1. Operational capacity increased substantially: mean daily HaH admissions increased from 2.11/day (59 admissions across 28 days) to 6.06/day (188 admissions across 31 days), corresponding to a rate ratio of 2.88 (95% CI 2.15–3.86; *p* < 0.001). This rate ratio was .calculated from the ratio of the two admission rates: 188/31 divided by 59/28. Bed capacity increased from 12 to 40 active HaH beds, clinical disciplines expanded from 2 to 10, and service coverage expanded from a 30-km radius to nationwide coverage supported by external nursing services. Patients admitted during wartime were younger than those admitted pre-war (63.1 ± 20.6 vs. 71.5 ± 16.4 years; *p* = 0.002), and the clinical case mix broadened. Therefore, unadjusted comparisons of clinical safety indicators should be interpreted descriptively rather than as evidence of equivalence. The proportion of urgent cases was similar between periods (85.6% vs. 84.7%; *p* = 0.835). Median length of stay was 3 days in both periods (IQR 2–5 vs. 2–5; *p* = 0.295). Transfers back to hospital during the HaH episode were observed in 4/59 (6.8%) pre-wartime admissions and 4/188 (2.1%) wartime admissions (*p* = 0.095). Repeat hospital admission within 14 days after HaH discharge, assessed through mid-April 2026, occurred in 5/59 (8.5%) and 16/188 (8.5%) admissions, respectively (*p* = 1.00). No deaths occurred during active HaH care in either period.

**Table 1:** Compared metrics of pre-war and war-time periods.

Metric	Pre-wartime baseline Feb 1–28, 2026	Wartime surge period Mar 1–31, 2026	*P* value/comparison
HaH admissions, n	59	188	—
Mean daily HaH admissions	2.11/day	6.06/day	Rate ratio 2.88; 95% CI 2.15–3.86; *p* < 0.001
Active HaH bed capacity, n	12	40	—
Additional nurses recruited, n	—	30	—
Geographic coverage	Approximately 30-km radius	Nationwide coverage with external nursing support	—
Clinical disciplines served, n	2	10	—
Age, years; mean (SD)	71.5 (16.4)	63.1 (20.6)	*p* = 0.002
Urgent cases, *n* (%)	50 (84.7%)	161 (85.6%)	*p* = 0.835
Length of stay, days; median (IQR)	3 (2–5)	3 (2–5)	*p* = 0.295
Transfers back to hospital during HaH, *n* (%)	4 (6.8%)	4 (2.1%)	*p* = 0.095
Repeat hospital admission within 14 days after HaH discharge, *n* (%)	5 (8.5%)	16 (8.5%)	*p* = 1.00
Mortality during active HaH care, *n* (%)	0 (0.0%)	0 (0.0%)	—

HaH, Hospital at Home; IQR, interquartile range; SD, standard deviation. Mortality during active HaH care was defined as death occurring while the patient was enrolled in HaH. Deaths after transfer back to conventional hospital care were reviewed separately and were not included in this table metric.

## Discussion

During the wartime period in March 2026, our HaH service underwent rapid operational scale-up. The strongest finding of this study is the demonstrated expansion of capacity, reflected by a nearly three-fold increase in mean daily admissions, increased bed capacity, broader geographic coverage, and inclusion of additional clinical disciplines. Short-term clinical safety indicators were reassuring, but because the wartime cohort was younger and clinically more heterogeneous, these outcomes should be interpreted as descriptive and unadjusted rather than as proof of clinical equivalence.

Our findings add a crisis-readiness perspective to the established HaH evidence base. Prior research has focused primarily on routine operations in stable settings, demonstrating that admission avoidance HaH can offer similar clinical outcomes to inpatient care and may reduce iatrogenic complications and costs in selected populations (1, 14). More recently, technology-enabled “virtual ward” and telemedicine-intensive HaH models have expanded, but the evidence remains heterogeneous with variability in intervention components, staffing structures, and monitoring intensity (15). Our wartime experience suggests that, beyond serving as a patient-centered alternative to hospitalization, telemedicine-controlled HaH can function as a rapid decompression valve for protected inpatient capacity when hospitals must simultaneously reduce exposure risk and maintain acute care throughput, with stable clinical performance measures.

From an operational standpoint, the Sheba BEYOND surge response maps onto core surge-capacity domains. First, it created space by substituting home-based care for a subset of patients who would otherwise occupy inpatient beds at a time when physical wards required relocation and consolidation. Second, it reconfigured staffing through rapid workforce expansion and use of external nursing resources coordinated by a centralized clinical team, consistent with surge guidance that emphasizes flexible staffing and rapid competency alignment. Third, it strengthened systems for triage, coordination, and escalation, all are elements repeatedly identified as necessary for safe HaH and for virtual care models more generally (16, 17).

The clinical indicators require cautious interpretation. No statistically significant increase was observed in transfers back to hospital, repeat hospital admission within 14 days, mortality during active HaH care, or length of stay. However, the wartime cohort was substantially younger and included a broader mixture of clinical disciplines than the pre-wartime cohort. These differences may confound the observed safety indicators and limit causal inference. In particular, the lower observed transfer-back rate during wartime may reflect differences in age, patient selection, clinical discipline, or other unmeasured case-mix factors rather than a true improvement in clinical performance. Accordingly, the data support feasibility of rapid scale-up with reassuring short-term safety signals, but they do not establish clinical equivalence between periods.

The wartime setting also highlighted staff-related implementation issues that are not captured by conventional clinical outcomes. Field teams continued home visits during missile attacks and required practical support for protected travel, real-time communication, and sheltering during alerts. This experience emphasized that future HaH preparedness models for conflict or disaster settings should include staff-safety procedures alongside patient-selection criteria, escalation protocols, and workforce-expansion plans. The willingness of staff to continue caring for patients under these conditions was essential to the service's continuity and should be explicitly considered in future models of crisis-responsive HaH care.

## Conclusions

In a high-threat wartime environment requiring rapid relocation of inpatients to fortified facilities, a telemedicine-led HaH service was rapidly expanded in volume, geographic coverage, workforce, bed capacity, and clinical scope. The principal evidence from this study supports operational feasibility and rapid scalability. Short-term clinical safety indicators were reassuring, but because of differences in age and case mix between periods, they should be interpreted as descriptive rather than confirmatory evidence of clinical equivalence. Health systems operating in disaster-prone or conflict settings may consider HaH readiness as part of preparedness planning, including predefined eligibility criteria, scalable staffing models, contracted home-visit capacity, standardized escalation pathways, staff-safety procedures, and interoperable telemedicine infrastructure. Future work should include adjusted analyses, stratification by clinical discipline, longer follow-up, and reproducible implementation playbooks for rapid HaH scale-up during crises.

## Limitations

This manuscript describes a single-center operational transformation and is therefore subject to limited generalizability. The one-month pre-wartime comparator period was short and may not fully represent usual seasonal or operational variability. The absence of randomization, the relatively small number of clinical outcome events, the selected patient population, and differences in case mix between periods limit causal inference regarding clinical effectiveness during wartime. Most importantly, the wartime group was significantly younger and included an expanded and more heterogeneous clinical case mix, making unadjusted clinical comparisons difficult to interpret. The existing Sheba BEYOND infrastructure may also limit generalizability to institutions without a mature virtual-hospital platform. Finally, the heterogeneous mix of clinical disciplines expanded during the surge complicates aggregation of outcomes and underscores the need for condition-specific reporting, standardized HaH component descriptions, adjusted analyses, longer follow-up, and quantitative assessment of patient experience.

### Strengths

This manuscript has several strengths. First, it provides a real-world description of rapid HaH scale-up during an active armed conflict, an implementation context that is rarely reported in detail. Second, it combines operational capacity metrics with short-term clinical safety indicators, allowing readers to distinguish evidence of scalability from clinical outcome observations. Third, the report documents concrete implementation components, including workforce expansion, bed-capacity growth, geographic expansion, external nursing integration, governance, logistics, and escalation pathways. Finally, the experience offers practical lessons for health systems considering HaH as part of preparedness planning for crises that constrain physical hospital capacity.

## Data Availability

The raw data supporting the conclusions of this article will be made available by the authors, without undue reservation.
